# Association Between Cdyn‐Guided PEEP and Nursing Activities During PACU Recovery in Infants Undergoing One‐Lung Ventilation: A Retrospective Observational Cohort Study

**DOI:** 10.1002/nop2.70935

**Published:** 2026-10-13

**Authors:** Mingran Hao, Bin Zhang, Yuejiao Han, Changbin Zhao, Zhaoyuan Dong, Benqin Song

**Affiliations:** ^1^ Department of Infectious Diseases Qilu Hospital of Shandong University Jinan China; ^2^ Department of Anesthesiology Children's Hospital Affiliated to Shandong University Jinan China

**Keywords:** anaesthesia recovery room, lung dynamic compliance, nursing workload, one‐lung ventilation, positive end‐expiratory pressure

## Abstract

**Aim:**

To evaluate the association between Cdyn‐guided PEEP during one‐lung ventilation (OLV) and nursing activities in the post‐anaesthesia care unit (PACU) among infants undergoing thoracoscopic surgery under general anaesthesia:

**Design:**

Retrospective observational cohort study.

**Methods:**

A retrospective analysis was conducted on clinical data from infants with congenital pulmonary airway malformation (CPAM) who underwent thoracoscopic lobectomy at a Children's Hospital Affiliated to Shandong University between January 2022 and February 2025. Patients were classified according to the recorded intraoperative PEEP strategy actually delivered during OLV: Group P was managed with Cdyn‐guided individualized PEEP; Group C was managed with fixed PEEP of 5 cmH_2_O. The primary outcome was PACU Nursing Activities Score (NAS). Baseline clinical characteristics, postoperative outcomes (incidence of delirium, pain scores), PACU observation time, and adverse events were compared between the two groups.

**Results:**

A total of 93 patients were included. Baseline characteristics were generally balanced, with most standardized mean differences < 0.2. After adjustment for prespecified covariates, Cdyn‐guided PEEP was associated with a lower NAS score (adjusted mean difference = −3.75; 95% CI: −6.44 to −1.07; *p* = 0.007). Compared with Group C, Group P was also associated with a shorter PACU stay and a lower composite adverse event rate.

**Patient or Public Contribution:**

No Patient or Public Contribution.

## 1 Introduction

The application of one‐lung ventilation (OLV) in thoracoscopic surgery facilitates precise lesion delineation and enhances operative space (McLaughlin et al. 2024). However, as a non‐physiological ventilation mode, OLV carries inherent risks of ventilation‐perfusion (V/Q) mismatch, hypoxemia, and lung injury (Ferrando et al. 2024). These intraoperative challenges necessitate intensive postoperative monitoring and management in the post‐anaesthesia care unit (PACU), a critical transitional environment for emergence and initial recovery (Templeton, Piccioni, and Chatterjee 2021). Optimizing ventilator settings is crucial to mitigate OLV‐related risks. Dynamic lung compliance (Cdyn), a key physiological parameter reflecting pulmonary tissue elasticity and airway resistance, has been employed to guide positive end‐expiratory pressure (PEEP) settings within lung‐protective ventilation strategies for OLV (Zorrilla‐Vaca et al. 2022). A fixed PEEP of 5 cmH_2_O is a conventional choice in anaesthetic ventilation management, used to maintain basic alveolar recruitment. While Cdyn‐guided individualized PEEP titration theoretically improves lung mechanics and gas exchange, its association with PACU recovery, especially on nursing workload and turnover efficiency, has not been well studied in infants. This retrospective observational cohort study evaluated the association between Cdyn‐guided PEEP and fixed PEEP during OLV and PACU outcomes, with PACU NAS as the primary outcome. We aimed to estimate the adjusted between‐group difference in NAS and to explore secondary outcomes including oxygenation, extubation time, PACU stay, and recorded adverse events.

## 2 Materials and Methods

This retrospective observational cohort study included 102 infants with congenital pulmonary airway malformation (CPAM) who underwent elective thoracoscopic lobectomy (including anatomical segmentectomy or wedge resection) at Children's Hospital Affiliated to Shandong University between January 2022 and February 2025. The study was approved by the Ethics Committee of children's Hospital Affiliated to Shandong University (approval number: SDFE‐IRB/P‐2024052). Patients were categorized into two groups based on their recorded intraoperative positive end‐expiratory pressure (PEEP) management during one‐lung ventilation (OLV): Group P (P for Protocol‐guided PEEP), which received individualized PEEP titration guided by dynamic lung compliance (Cdyn), and Group C (C for Conventional PEEP), which received a fixed PEEP of 5 cmH_2_O. The choice of strategy reflected anesthesiologist preference. No randomization was performed. Patients were classified according to the strategy actually delivered, not intended allocation. The target estimand was the adjusted mean difference in PACU NAS between infants actually managed with Cdyn‐guided PEEP and those managed with fixed PEEP, conditional on prespecified baseline and surgical covariates. The primary outcome was PACU NAS.

### 2.1 Inclusion Criteria

Age 0–12 months; Weight‐for‐length within the 5th to 95th percentiles according to the World Health Organization (WHO) Child Growth Standards; American Society of Anesthesiologists (ASA) physical status I–II; Administration of either Cdyn‐guided PEEP titration or a fixed PEEP of 5 cm H_2_O during OLV. Exclusion Criteria: Preterm birth (gestational age < 37 weeks); Asthma or active respiratory/lung infections within the past 4 weeks; Prior lung surgery; Neuromuscular disorders; Preoperative use of analgesics or sedatives within one week prior to surgery; Neurodevelopmental disorders (e.g., intellectual disability, autism spectrum disorder), cerebral palsy, central nervous system disorders, or metabolic diseases；Intraoperative conversion to open thoracotomy; Intraoperative transfusion of red blood cell products; Severe intraoperative complications (e.g., massive haemorrhage, anaphylactic shock); Incomplete data; Postoperative transfer to the Intensive Care Unit (ICU). This criterion includes both direct transfer from the operating room and delayed transfer from the PACU. ICU transfer is determined by clinical need (e.g., hemodynamic instability, vasopressor support, refractory hypoxemia, or prolonged mechanical ventilation).

### 2.2 Anaesthetic Protocol

Fasting Protocol: 6 h for solid food/infant formula, 4 h for breast milk, and 2 h for clear fluids. Standard monitoring: ECG, SpO_2_, HR, BP, temperature, and end‐tidal CO_2_ (PetCO_2_). Induction: Propofol (2–3 mg/kg), cisatracurium (0.15–0.2 mg/kg), and sufentanil (0.1–0.5 μg/kg). Bronchial blocker (Tappa EBT0105, China) placement under video laryngoscopy, followed by orotracheal intubation (Tuoren, China). Radial artery cannulation for invasive BP monitoring. Continuous infusion: Propofol (8~12 mg kg^−1^·h^−1^) and remifentanil (0.2~0.3 μg·kg^−1^·min^−1^).

After all children completed tracheal intubation, their position was adjusted to the lateral position. Bilateral lung volume‐controlled ventilation (VCV) was delivered via the anaesthesia machine (Maquet Flow‐iC20, Sweden): inspired oxygen concentration (FiO2) 0.5, fresh gas flow 2 L/min, tidal volume (VT) 8 mL/kg, inspiratory/expiratory ratio (I:E) = 1:2, PEEP = 0 cmH2O. After 15 min of bilateral ventilation, all children were observed under bronchoscopy to adjust the position of the bronchial blocker (BB) balloon at the opening of the isolated‐side bronchus. After inflating the balloon, unilateral lung volume‐controlled ventilation (OLV‐VCV) was performed: FiO2 0.5, fresh gas flow 2 L/min, VT 6 mL/kg, I:E = 1:2.

A stepwise PEEP titration was used for lung recruitment (Young et al. 2019). After lung recruitment, PEEP settings were established. The P group started with PEEP = 0 cmH2O and increased by 1 cmH2O after 10 respiratory cycles, up to a maximum of 10 cmH2O, recording the Cdyn at each PEEP level, calculated as VT / (Ppeak − PEEP). The minimum PEEP level at which maximum Cdyn was achieved was used until the end of OLV. The C group used a fixed PEEP of 5 cmH2O throughout OLV. At the end of OLV, the BB balloon was deflated, and both the C group and P group returned to the bilateral ventilation parameters established before OLV.

After the procedure, neostigmine 0.02 mg/kg and atropine 0.01 mg/kg were promptly administered to antagonize residual muscle relaxants. When the child's tidal volume was ≥ 6 mL/kg and the respiratory rate was ≥ 15 breaths/min, the endotracheal tube was extubated and the child was sent to the PACU, where they were monitored until the recovery period, achieving a modified Aldrete score of ≥ 9 before being returned to the ward. The participant screening, enrollment, and group allocation process is summarized in Figure 1.

**FIGURE 1:**
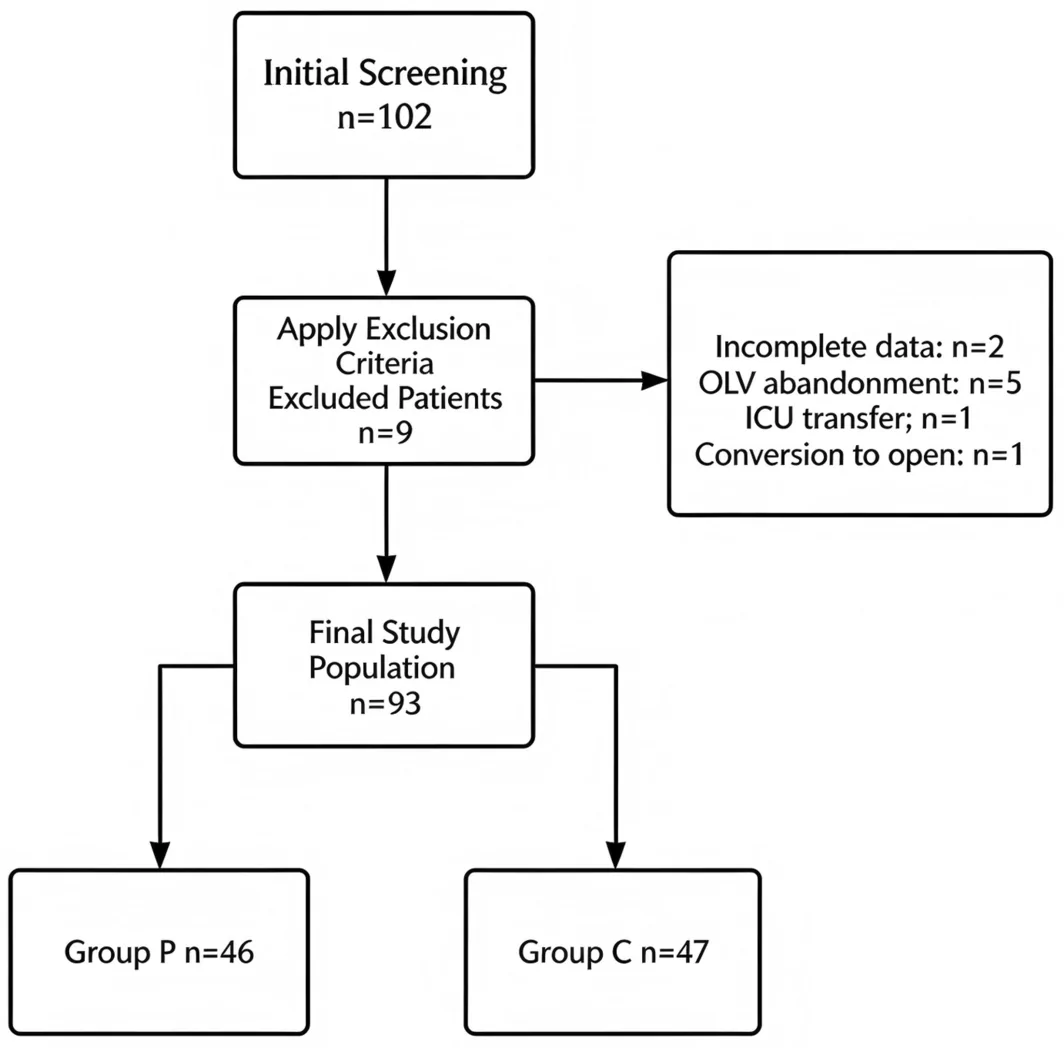
Participant flow diagram. Of 102 screened infants, 9 were excluded (incomplete data, *n* = 2; OLV abandonment, *n* = 5; conversion to thoracotomy, *n* = 1; ICU transfer, *n* = 1), leaving 93 analysed (Group P, *n* = 46; Group C, *n* = 47). ICU, intensive care unit; OLV, one‐lung ventilation.

### 2.3 Data Collection

Compare the general clinical data of the two groups of patients, including sex, age in months, BMI, ASA classification, lesion location, and duration of OLV. Compare intraoperative monitoring parameters for the patients, including mean arterial pressure (MAP), heart rate (HR), plateau pressure (Pplat), peak airway pressure (Ppeak), extubation time (from the end of the surgery to the successful removal of the endotracheal tube), dynamic compliance (Cdyn), P/F ratio (PaO_2_/FiO_2_ ratio), and the difference in partial pressure of oxygen between alveolar and arterial blood (P(A‐a)O_2_) at four time points: 15 min after lateral position bilateral ventilation (T1), 30 min after PEEP setting (T2), 60 min after PEEP setting (T3), and 15 min after returning to bilateral ventilation in the lateral position (T4).

NAS was extracted from routine PACU nursing records, prospectively recorded by attending PACU nurses throughout the PACU stay. All 23 NAS items were applicable, with no modifications or missing data. Scoring followed the standard institutional protocol. Assess and compare the results of delirium and pain in the two groups of children, using the Paediatric Anaesthesia Emergence Delirium (PAED) scale and the FLACC scale for evaluation (Cai et al. 2024):For FLACC ≥ 4 and PAED < 10, 0.2 mg/kg of pentazocine will be administered for pain management. If PAED ≥ 10 and FLACC < 4, initial comfort measures will be implemented, and if ineffective, 1 mg/kg of propofol will be administered. If a child exhibits both delirium and pain (PAED ≥ 10, FLACC ≥ 4), priority will be given to pain management. Lastly, compare the PACU length of stay and the occurrence of adverse events (laryngospasm, coughing, hypoxemia (SpO_2_ < 90% or PaO_2_ < 60 mmHg), nausea and vomiting, bradycardia) during the PACU period between the two groups of children.

### 2.4 Statistical Analysis

Statistical analysis was performed using SPSS 23.0. Continuous variables are presented as mean (SD) or median (IQR), and categorical variables as *n* (%). Baseline balance was assessed using standardized mean differences. The primary outcome was PACU NAS, and the primary analysis was an adjusted linear regression model including prespecified covariates (age, sex, BMI, ASA physical status, lesion laterality, and OLV duration). Unadjusted *t*‐tests were used for descriptive purposes only. Repeated‐measures ANOVA was used for intraoperative parameters, with group × time interaction as the principal inferential test; Greenhouse–Geisser correction was applied when sphericity was violated. Categorical outcomes were compared using the chi‐square test, continuity‐corrected chi‐square test, or Fisher's exact test. Subgroup analyses used interaction terms. All tests were two‐tailed, and *p* < 0.05 was considered statistically significant.

## 3 Results

A total of 102 paediatric patients were initially identified and all received one of the two PEEP strategies during OLV. After exposure, 9 patients were excluded: incomplete intraoperative data (*n* = 2), intraoperative OLV abandonment (*n* = 5), conversion to thoracotomy (*n* = 1), and postoperative ICU transfer (*n* = 1). Finally, 93 patients were analysed: 46 in the P group and 47 in the C group. Baseline characteristics are shown in Table 1.

**TABLE 1:** Baseline characteristics and PEEP titration results.

Parameters	P group (*n* = 46)	C group (*n* = 47)	SMD	*p*
Sex (male)	28 (60.9%)	24 (51.1%)	0.20	0.341^a^
Age (months)	8.26 (2.07)	8.74 (1.97)	0.24	0.252
BMI	17.48 (1.00)	17.41 (1.25)	0.06	0.761
Lesion location (left)	29 (63.0%)	26 (55.3%)	0.16	0.574^a^
ASA				
I	5 (10.9%)	2 (4.3%)		
II	41 (89.1%)	45 (95.7%)	0.25	0.665^b^
OLV time (min)	83.24 (14.17)	81.47 (13.15)	0.13	0.534
Total operation time (min)	90.20 (13.96)	88.55 (12.98)	0.12	0.558
Anaesthesia time (min)	109.54 (13.22)	113.94 (16.47)	0.29	0.160
Total infused fluid (ml)	125.87 (18.66)	115.26 (18.88)	0.56	0.093
PEEP Titration Results (cmH_2_O)	7.13 (1.26)	5.00 (0.00)	—	< 0.001

*Note:* Data are presented as mean (SD) or *n* (%). *p* values were calculated using the independent *t*‐test, chi‐square test, or Fisher's exact test, as appropriate.

^a^ Pearson's chi‐square test.

^b^ Chi‐square test with continuity correction.

Repeated‐measures ANOVA was used to compare intraoperative parameters between groups over time, with group × time interaction as the principal inferential test. Significant group × time interactions were found for Cdyn (*p* < 0.001), P/F ratio (*p* = 0.003), and P(A‐a)O_2_ (*p* = 0.006), but not for HR, MAP, or Pplat (all *p* > 0.05). Post hoc comparisons showed that Cdyn was higher in Group P at T2 and T3 (both *p* < 0.001), P/F ratio was higher at T2, T3, and T4 (*p* = 0.020, 0.019, and 0.001, respectively), and P(A‐a)O_2_ was lower at T2, T3, and T4 (*p* = 0.001, 0.008, and 0.001, respectively). (Table 2).

**TABLE 2:** Comparison of Intraoperative Parameters Between Groups over Time.

Parameter	Time point	Group P (*n* = 46), mean (SD)	Group C (*n* = 47), mean (SD)	Between‐group difference (*P* − C) (95% CI)	*p*
HR (beats/min)	T1	114.87 (6.59)	117.11 (7.21)	—	—
	T2	121.20 (6.92)	119.55 (4.88)	—	—
	T3	118.37 (5.93)	119.46 (4.10)	—	—
	T4	121.63 (6.35)	121.45 (7.00)	—	—
	Effect	Group: *F*(1, 91) = 0.170, *p* = 0.681	Time: F(2.625, 238.908) = 20.991, *p* < 0.001	G × T: *F*(2.625, 238.908) = 2.548, *p* = 0.064	
MAP (mmHg)	T1	58.63 (3.69)	58.34 (5.02)	—	—
	T2	54.33 (3.25)	55.21 (4.56)	—	—
	T3	57.67 (3.93)	57.43 (3.87)	—	—
	T4	53.83 (4.13)	52.87 (3.87)	—	—
	Effect	Group: *F*(1, 91) = 0.053, *p* = 0.818	Time: *F*(2.620, 238.409) = 60.358, *p* < 0.001	G × T: *F*(2.620, 238.409) = 1.543, *p* = 0.209	
Pplat (cmH_2_O)	T1	13.43 (1.44)	13.06 (1.34)	—	—
	T2	22.63 (1.45)	22.98 (1.38)	—	—
	T3	23.33 (1.98)	24.00 (1.64)	—	—
	T4	12.89 (1.82)	13.57 (2.09)	—	—
	Effect	Group: *F*(1, 91) = 2.572, *p* = 0.112	Time: *F*(3, 273) = 1321.514, *p* < 0.001	G × T: *F*(3, 273) = 2.422, *p* = 0.066	
Cdyn (mL/cmH_2_O)	T1	8.06 (0.70)	7.76 (0.74)	0.295 (−0.002 to 0.591)	0.051
	T2	4.67 (0.60)	3.78 (0.38)	0.891 (0.684 to 1.097)	< 0.001
	T3	4.64 (0.61)	3.70 (0.43)	0.933 (0.715 to 1.150)	< 0.001
	T4	7.90 (0.69)	7.67 (0.78)	0.236 (−0.068 to 0.540)	0.126
	Effect	Group: *F*(1, 91) = 45.119, *p* < 0.001	Time: *F*(1.922, 174.859) = 1413.021, *p* < 0.001	G × T: *F*(1.922, 174.859) = 11.118, *p* < 0.001	
P/F ratio (mmHg)	T1	370.91 (44.63)	376.75 (47.10)	−5.843 (−24.752 to 13.066)	0.541
	T2	271.97 (38.56)	253.98 (34.94)	17.993 (2.842 to 33.144)	0.020
	T3	276.23 (39.24)	255.52 (43.88)	20.702 (3.543 to 37.861)	0.019
	T4	333.47 (40.92)	299.10 (50.74)	34.366 (15.354 to 53.378)	0.001
	Effect	Group: *F*(1, 91) = 7.207, *p* = 0.009	Time: *F*(2.375, 216.102) = 206.226, *p* < 0.001	G × T: *F*(2.375, 216.102) = 5.293, *p* = 0.003	
P(A‐a)O_2_ (mmHg)	T1	76.14 (16.54)	75.38 (17.92)	0.758 (−6.350 to 7.866)	0.833
	T2	132.50 (18.92)	151.33 (32.32)	−18.825 (−29.764 to −7.887)	0.001
	T3	144.48 (18.09)	156.07 (22.89)	−11.583 (−20.094 to −3.071)	0.008
	T4	82.16 (15.58)	92.40 (13.89)	−10.239 (−16.315 to −4.163)	0.001
	Effect	Group: *F*(1, 91) = 13.834, *p* < 0.001	Time: *F*(2.445, 222.462) = 405.862, *p* < 0.001	G × T: *F*(2.445, 222.462) = 4.661, *p* = 0.006	

*Note:* Data are presented as mean (SD). Repeated‐measures ANOVA (group as between‐subjects factor, time as within‐subjects factor) was used, with the group × time interaction as the principal inferential test. Greenhouse–Geisser correction was applied for HR, MAP, Cdyn, P/F ratio, and P(A‐a)O_2_ due to sphericity violation; Pplat met sphericity (*p* = 0.068). For variables with significant group × time interaction, between‐group differences (Group *P* − Group C) and Sidak‐adjusted *p* values are reported; negative values indicate lower values in Group P. Post hoc comparisons were exploratory. HR, heart rate; MAP, mean arterial pressure; Pplat, plateau pressure; Cdyn, dynamic lung compliance; P/F ratio, PaO_2_/FiO_2_ ratio; P(A‐a)O_2_, alveolar–arterial oxygen gradient; T1–T4, 15 min after lateral position bilateral ventilation, 30 min after PEEP setting, 60 min after PEEP setting, and 15 min after returning to bilateral ventilation, respectively.

No statistically significant differences were observed between the two groups in maximum PAED (Paediatric Anaesthesia Emergence Delirium) scores, propofol rescue rates, pain incidence, or pentazocine rescue requirements during PACU stay (all *p* > 0.05). For the composite adverse event outcome, Fisher's exact test showed a lower event rate in Group P than in Group C (3/46 [6.5%] vs. 11/47 [23.4%]; risk difference = −16.9%, 95% CI: −31.0% to −2.8%; OR = 0.23, 95% CI: 0.06–0.88; Fisher's exact *p* = 0.040). (Table 3).

**TABLE 3:** Postoperative recovery outcomes and adverse events in PACU.

Outcome	Group P (*n* = 46)	Group C (*n* = 47)	Effect estimate (95% CI)	OR (95% CI)	*p*
*Continuous outcomes, mean (SD)*			*Mean difference (95% CI)*		
Max PAED score	7.26 (1.74)	7.40 (1.97)	−0.14 (−0.91 to 0.63)	—	0.328
Extubation time (min)	21.93 (5.49)	25.19 (6.14)	−3.26 (−5.68 to −0.84)	—	0.008
PACU stay time (min)	37.63 (4.92)	42.89 (5.65)	−5.26 (−7.45 to −3.07)	—	< 0.001
Postoperative hospital stay (days)	6.50 (1.34)	7.64 (1.75)	−1.14 (−1.79 to −0.49)	—	0.082
*Binary outcomes, n (%)*			*Risk difference (95% CI)*		
Propofol rescue	2 (4.35)	4 (8.51)	−4.16% (−13.2 to 4.8)	0.49 (0.09–2.81)	0.693^b^
Pain incidence	8 (17.39)	10 (21.28)	−3.89% (−19.4 to 11.6)	0.78 (0.28–2.16)	0.635^a^
Pentazocine rescue	7 (15.22)	9 (19.15)	−3.93% (−18.4 to 10.6)	0.76 (0.26–2.22)	0.615^a^
*Adverse events*					
Laryngospasm	1 (2.17)	1 (2.13)	Not estimable	Not estimable	> 0.99
Coughing	0 (0)	3 (6.38)	Not estimable	Not estimable	0.248^c^
Hypoxemia	1 (2.17)	6 (12.77)	Not estimable	Not estimable	0.111^c^
Nausea/Vomiting	1 (2.17)	1 (2.13)	Not estimable	Not estimable	> 0.99
Bradycardia	0 (0)	0 (0)	Not estimable	Not estimable	Not estimable
*Total adverse events* ^1^	3 (6.52)	11 (23.40)	−16.88% (−31.0 to −2.8)	0.23 (0.06–0.88)	0.040^c^

Abbreviations: CI, confidence interval; OR, odds ratio.

^1^ Composite adverse event was defined a priori as any of laryngospasm, coughing, hypoxemia, nausea/vomiting, or bradycardia. Each child contributed once (patients with ≥ 1 event). Data are presented as mean (SD) or *n* (%). *p* values were calculated using the independent *t*‐test, chi‐square test, or Fisher's exact test, as appropriate.

^a^ Pearson's chi‐square test.

^b^ Chi‐square test with continuity correction.

^c^ Fisher's exact test constant.

In the unadjusted analysis, the NAS score was lower in Group P than in Group C (mean difference = −3.89 points; 95% CI: −6.53 to −1.25; *p* = 0.004). After adjustment for age, sex, BMI, ASA physical status, lesion laterality, and OLV duration, the adjusted mean difference remained statistically significant (−3.75 points; 95% CI: −6.44 to −1.07; *p* = 0.007). ASA physical status (*B* = 5.87, *p* = 0.030) and sex (*B* = −2.77, *p* = 0.049) were also significantly associated with NAS. The overall model was statistically significant (*F* = 2.441, *p* = 0.025, adjusted *R* ^2^ = 0.099), and no problematic collinearity was observed (all VIF < 1.15). (Table 4).

**TABLE 4:** Comparison of Nursing Activities Score (NAS) between groups: unadjusted and adjusted analyses.

Model	Group P (*n* = 46)	Group C (*n* = 47)	Mean difference (*P* − C) (95% CI)	*p*
Unadjusted	24.10 (4.75)	27.99 (7.71)	−3.89 (−6.53, −1.25)	0.004
Adjusted^a^	—	—	−3.75 (−6.44, −1.07)	0.007

Abbreviations: CI, confidence interval; NAS, Nursing Activities Score; OLV, one‐lung ventilation; SD, standard deviation.

^a^ Adjusted for age, sex, BMI, ASA physical status, lesion laterality, and OLV duration. Data are presented as mean (SD) for the unadjusted model. Mean difference = Group P minus Group C; negative values indicate lower NAS in Group P.

Exploratory subgroup analyses were performed for age, lesion location, and OLV duration. The interaction terms were not statistically significant for age (*p* = 0.808), lesion location (*p* = 0.636), or OLV duration (*p* = 0.132). Because the interactions were not significant and the study was underpowered for subgroup analyses, subgroup‐specific adjusted mean differences were not estimated. Subgroup sample sizes and NAS medians (IQR) are presented in Table 5 for descriptive purposes. (Table 5).

**TABLE 5:** Subgroup analyses of the association between Cdyn‐guided PEEP and Nursing Activities Score.

Subgroup	Group P, *n*	Group P NAS, median (IQR)	Group C, *n*	Group C NAS, median (IQR)	*P* for interaction
Overall	46	20.70 (20.70–26.20)	47	26.20 (20.70–37.50)	—
Age					0.808
≤ 9 months	31	20.70 (20.70–26.20)	31	26.30 (20.70–37.50)	
> 9 months	15	23.66 (21.07–26.17)	16	22.90 (20.70–36.40)	
Lesion location					0.636
Left lung	29	20.70 (20.70–26.20)	26	26.20 (20.70–36.53)	
Right lung	17	20.70 (20.70–26.20)	21	26.20 (20.70–38.10)	
OLV duration					0.132
≤ 80 min	23	20.70 (20.70–26.20)	25	25.10 (20.70–35.30)	
> 80 min	23	20.70 (20.70–26.20)	22	28.45 (20.70–37.80)	

*Note:* Data are presented as median (IQR) for descriptive purposes only.

Abbreviations: IQR, interquartile range; NAS, Nursing Activities Score; OLV, one‐lung ventilation.

## 4 Discussion

Infants possess unique physiological challenges during thoracic surgery, including smaller body size, reduced functional residual capacity, lower oxygen reserves, and diminished chest wall elasticity, which predispose them to hypoxemia, hypercapnia, ventilation/perfusion (V/Q) mismatch, and intrapulmonary shunting during one‐lung ventilation (OLV) (Trachsel et al. 2016). Conventional intraoperative interventions to improve oxygenation—such as increasing inspired oxygen concentration, respiratory rate, tidal volume, or airway pressure—may exacerbate lung injury and elevate the risk of adverse events during post‐anaesthesia care unit (PACU) recovery (Templeton, Miller, et al. 2021). Congenital pulmonary airway malformation (CPAM), characterized by adenomatous overgrowth of terminal bronchioles and often involving unilateral lung segments or lobes, accounts for approximately 40% of paediatric thoracoscopic lobectomies (Pederiva et al. 2023). This study focused on infants aged 0–12 months undergoing OLV for CPAM resection. Bronchial blockers (BB) were placed outside the lumen of the endotracheal tube in all children. Our analysis found that the use of Cdyn‐guided individualized PEEP during OLV was associated with better intraoperative oxygenation, shorter extubation time, shorter PACU stay, and fewer recorded postoperative adverse events compared with fixed PEEP. These associations may be related to shorter PACU stay and lower NAS.

PEEP, a cornerstone of lung‐protective ventilation, maintains alveolar recruitment and increases functional residual capacity. However, fixed PEEP levels may inadequately address heterogeneous pathophysiological states, potentially upregulating alveolar epithelial injury biomarkers and inflammatory cascades, thereby contributing to ventilator‐induced lung injury (VILI) (Wong et al. 2024; Beitler et al. 2019; Dianti et al. 2022). Dynamic lung compliance (Cdyn), reflecting alveolar recruitment, lung elasticity, airway resistance, and intrathoracic pressure, served as the physiological target for individualized PEEP optimization in Group P. Compared to Group C (fixed PEEP), Group P exhibited comparable hemodynamic stability (HR and MAP at T1–T4, *p* > 0.05) but superior P(A‐a)O_2_ (*p* < 0.05) and higher Cdyn (*p* < 0.05) at critical intraoperative phases (T2–T4). Although Cdyn served as the titration target for PEEP in Group P, higher Cdyn values may, on physiological grounds, be associated with independent indicators of clinical benefit. These benefits likely stem from improved alveolar homogeneity and reduced V/Q mismatch, aligning with findings by Li et al. (2022)., who reported enhanced respiratory mechanics and attenuated inflammation with Cdyn‐guided PEEP in laparoscopic surgery.

PACU, a critical hub for postoperative monitoring and early recovery, demands meticulous nursing oversight for pain management, adverse event mitigation, and emergency intervention (Vishneski et al. 2021). While Group P showed numerically lower PAED scores, propofol/pentazocine rescue rates, and pain incidence, these differences lacked statistical significance (*p* > 0.05). However, Group P demonstrated a significantly lower overall adverse event rate (*p* < 0.05). This was observed concurrent with the application of intraoperative lung‐protective strategies and a reduced risk of VILI.

The NAS scale was proposed by Miranda et al. (2003) and validated in a study involving 99 ICUs across 15 countries. It serves as a tool for measuring nursing workload in the intensive care unit (ICU) and features a well‐developed and scientific scoring system. The scale closely integrates patient classification with nursing activities, with scores derived from 23 NAS items indicating the percentage of time nurses spend on direct care for critically ill patients over a 24‐h period. A total score of 100.0% represents the work of one nurse in 24 h, while the sum of the 23 items ranges from 0% to 177% (Nieri et al. 2018). In a multicenter study involving 235 patients, an examination and calculation of each nurse's NAS over 24 h found that the NAS scale could account for 75%–90% of the actual workload of ICU nurses (Stafseth et al. 2011). In this study, the NAS scale was used to summarize and assess the nursing workload for children in the PACU. After adjustment for prespecified covariates, Cdyn‐guided PEEP remained associated with lower NAS scores (adjusted mean difference = −3.75; 95% CI: −6.44 to −1.07; *p* = 0.007; Table 4). Although the MCID for NAS in the PACU is not established, this difference may suggest a lower observed nursing workload in Group P. The observed difference was associated with enhanced lung protection from Cdyn‐guided PEEP during OLV in Group P. This was accompanied by a lower incidence of adverse events and a corresponding reduction in nursing workload related to monitoring, positioning, medication, fluid management, and emergency resuscitation as captured by the NAS. Exploratory subgroup analyses revealed no statistically significant heterogeneity in the observed associations across the subgroups of age, lesion location, or OLV duration (all interaction *p*‐values > 0.05). When comparing the postoperative hospital stay between the two groups, the mean length of stay in Group P was about 1.1 days shorter than that in Group C. Although this trend did not reach statistical significance (*p* = 0.082), it may suggest that the shorter PACU stay in Group P did not come at the expense of prolonged overall hospitalization. Whether this difference is clinically meaningful, however, remains to be confirmed in larger studies. This consistency in treatment response underpins the potential applicability of Cdyn‐guided PEEP in enhancing PACU efficiency for infant thoracoscopic surgery.

## 5 Limitations

This study has several limitations. The retrospective, single‐center design and small sample size mean that residual and unmeasured confounding cannot be excluded. Post‐exposure exclusions (OLV abandonment, conversion to thoracotomy, and ICU transfer) may have introduced selection bias, although these events were reported as safety outcomes; a formal sensitivity analysis was not feasible because of the small number of events. The NAS was developed for ICU settings, and its validity in the infant PACU population remains uncertain; total NAS may also be influenced by PACU duration. Finally, subgroup analyses and comparisons of sparse adverse events were underpowered, and the 95% CI for the adjusted NAS difference was relatively wide. These findings should therefore be considered hypothesis‐generating and require confirmation in prospective studies.

## 6 Conclusion

In this retrospective cohort of infants with CPAM undergoing OLV, the use of Cdyn‐guided PEEP was associated with better intraoperative gas exchange and lung mechanics, fewer recorded PACU adverse events, and shorter PACU stay. The concomitant lower NAS scores may suggest a lower observed nursing workload during the recovery period. These findings are consistent with Cdyn‐guided PEEP as a potential component of lung‐protective ventilation, though its potential associations require confirmation in prospective studies.

## Author Contributions

**Mingran Hao:** conceptualization, methodology, data curation, investigation, funding acquisition, formal analysis, writing – review and editing, project administration. **Bin Zhang:** investigation, data curation, funding acquisition, writing – review and editing. **Yuejiao Han:** investigation, visualization, supervision. **Changbin Zhao:** investigation, visualization, validation. **Zhaoyuan Dong:** investigation, project administration, writing – review and editing. **Benqin Song:** conceptualization, methodology, data curation, investigation, funding acquisition, formal analysis, writing – review and editing, project administration.

## Funding

This work was supported by the Science and Technology Program of Jinan Municipal Health Commission (Grant 2024304018) and the Ji Nan Health High‐Caliber Talent Project (Grant 202512).

## Ethics Statement

As data was collected retrospectively, and all patients were de‐identified through being assigned a code, individual consent was not required. This study was approved by the Institutional Review Board (IRB) of Children's Hospital Affiliated to Shandong University (Approval No.: SDFE‐IRB/P‐2024052).

## Conflicts of Interest

The authors declare no conflicts of interest.

## Data Availability

The data that support the findings of this study are available from the corresponding author upon reasonable request, as they contain information that could compromise the privacy of research participants.
